# Outcomes of allogeneic haematopoietic stem cell transplantation for paediatric patients with MLL-rearranged acute myeloid leukaemia

**DOI:** 10.1186/s12885-022-09978-3

**Published:** 2022-08-16

**Authors:** Lu Bai, Yong-zhan Zhang, Chen-hua Yan, Yu Wang, Lan-ping Xu, Xiao-hui Zhang, Le-ping Zhang, Xiao-jun Huang, Yi-fei Cheng

**Affiliations:** 1 Department of Hematology, Peking University Institute of Hematology, Peking University People’s Hospital, Beijing, China; 2 Department of Pediatrics, Peking University People’s Hospital, Peking University, Beijing, China

**Keywords:** MLL rearrangement, Paediatric patient, Acute myeloid leukaemia, Haploidentical haematopoietic stem cell transplantation

## Abstract

**Background:**

The presence of mixed-lineage leukaemia rearrangement (MLL-r) in paediatric patients with acute myeloid leukaemia (AML) is a poor prognostic predictor. Whether allogeneic haematopoietic stem cell transplantation (allo-HSCT) is beneficial in such cases remains unclear.

**Methods:**

We evaluated the outcomes and prognostic factors of allo-HSCT in 44 paediatric patients with MLL-r AML in the first complete remission (CR1) between 2014 and 2019 at our institution.

**Results:**

For all the 44 patients, the 3-year overall survival (OS), event-free survival (EFS), and cumulative incidence of relapse (CIR) were 74.5%, 64.1%, and 29.1%, respectively. Among them, 37 (84.1%) patients received haploidentical (haplo)-HSCT, and the 3-year OS, EFS, and CIR were 73.0%, 65.6%, and 26.4%, respectively. The 100-day cumulative incidence of grade II–IV acute graft-versus-host disease (aGVHD) post-transplantation was 27.3%, and that of grade III–IV aGVHD was 15.9%. The overall 3-year cumulative incidence of chronic graft-versus-host disease (cGVHD) post-transplantation was 40.8%, and that of extensive cGVHD was 16.7%. Minimal residual disease (MRD)-positive (MRD +) status pre-HSCT was significantly associated with lower survival and higher risk of relapse. The 3-year OS, EFS, and CIR differed significantly between patients with MRD + pre-HSCT (*n* = 15; 48.5%, 34.3% and 59%) and those with MRD-pre-HSCT (*n* = 29; 89.7%, 81.4% and 11.7%). Pre-HSCT MRD + status was an independent risk factor in multivariate analysis.

**Conclusions:**

Allo-HSCT (especially haplo-HSCT) can be a viable strategy in these patients, and pre-HSCT MRD status significantly affected the outcomes.

## Background

With continuous advances in treatment strategies, the overall survival (OS) rate of paediatric acute myeloid leukaemia (AML) is almost 70% [1–4]. Nevertheless, the cure rates for different subtypes vary. The presence of MLL rearrangements (MLL-r), which are detected in 15%–20% of all patients with AML, predict an overall poor prognosis [2, 5, 6]. In a recent study involving a large cohort of paediatric patients, the long-term overall survival (OS) and event-free survival (EFS) were only 56% and 44%, respectively, with an association between different translocation partners and large differences in prognoses [6]. Therefore, there is room for advances in the current therapeutic strategies for paediatric MLL-r AML.

According to the European Leukaemia Net-2017 risk stratification, adult patients with MLL-r except t(9;11) were considered high-risk patients [7], and allogeneic haematopoietic stem cell transplantation (allo-HSCT) in the first complete remission (CR1) could lead to a more favourable outcome [8–10]. However, paediatric patients with MLL-r have been classified as intermediate-risk patients in some clinical trials and as high-risk patients in others. Considering the continuous advances in chemotherapy and transplantation-related toxicity effects, HSCT in CR1 is restricted to high-risk paediatric AML [3]. Therefore, it remains controversial whether allo-HSCT should be performed in paediatric MLL-r AML. Several studies have shown that allo-HSCT is a curative option and is beneficial in paediatric MLL-r AML, especially in the poor prognosis subgroups with MLL-ALL1-fused gene from chromosome 6 (MLL-AF6) or MLL-AF10 [6, 11]. However, due to their low incidence, the number of reported cases documenting the use of allo-HSCT are limited. Therefore, more studies are needed to improve the knowledge of allo-HSCT for this type of leukaemia.

This study aimed to explore the role of allo-HSCT (especially haploidentical [haplo]-HSCT) in the treatment of paediatric patients with MLL-r AML in CR1 and investigated the prognostic factors of these patients.

## Methods

### Patients

Between January 2014 and October 2019, 44 paediatric patients aged greater than or equal to 1 year and less than 18 years with MLL-r AML who received allogeneic HSCT in CR1 at our institution were enrolled into this study. The diagnosis of AML was made according to the Morphology, Immunology, Cytogenetics, and Molecular biology criteria [12], and the risk stratification was mainly based on cytogenetics, molecular features, and disease response [13, 14]. MLL-r was detected using one of three ways: karyotype analysis, fluorescence in situ hybridisation (FISH) test, or quantitative real-time polymerase chain reaction (qRT-PCR) [11, 15–17]. This clinical study was approved by the Ethics Committee of Peking University People’s Hospital and was carried out in accordance with the Declaration of Helsinki. Written informed consent for participation was obtained from the parents/guardians of all the patients.

### Chemotherapy before transplantation

The patients received an initial 8-day induction therapy consisting of cytarabine (150 mg/m^2^ on days 1–7), idarubicin (10 mg/m^2^ on days 3 and 5), and etoposide (100 mg/m^2^ on days 6–8). After two induction cycles, the subsequent consolidation chemotherapy was administered as previously reported [18], consisting of three regimens used alternately: AD (cytarabine 4 g/m^2^ on days 1–4 and idarubicin 10 mg/m^2^ on days 2 and 4), HA (cytarabine 150 g/m^2^ on days 1–7 and homoharringtonine 3 mg/m^2^ for days 1–7), and ADVP16 (cytarabine 150 mg/m^2^ on days 1–7, idarubicin 10 mg/m^2^ on days 3 and 5, and etoposide 100 mg/m^2^ on days 6–8). Patients with cKIT mutations were administered dasatinib (75 mg/m^2^/d) during chemotherapy intermission.

### Transplantation regimens

After two induction cycles and several cycles of consolidation therapy, all patients underwent allo-HSCT in CR1. Seven patients had human leukocyte antigen (HLA)-matched donors (one underwent matched unrelated donor transplant [MUDT], six underwent matched sibling donor transplant [MSDT]), and the remaining 37 patients had haploidentical donors (HIDs) and opted for haplo-HSCT. As reported previously [15, 18, 19], the conditioning regimens for haplo-HSCT and MUDT were essentially the same, consisting of cytarabine (4 g/m^2^/d) on days 9 and 10, busulfan (0.8 mg/kg/every 6 h) on days 6–8, cyclophosphamide (1.8 g/m^2^/d) on days 4–5, semustine (250 mg/m^2^) on day 3, and anti-thymocyte globulin (ATG) (2.5 mg/kg/d; Sang Stat, Lyon, France) on days 2–5. The conditioning regimen for MSDT was identical to that for haplo-HSCT without ATG. Cyclosporine A (CSA, intravenous infusion), mycophenolate mofetil (MMF, oral administration), and short-term methotrexate (MTX intravenous infusion) were administered to all patients for graft-versus-host disease (GVHD) prophylaxis. CSA was started on day − 3 at 2.5 mg/kg/d. CSA concentrations were monitored weekly and adjusted to maintain a trough concentration of 150 to 250 ng/mL. CSA dosage was decreased gradually in subjects without GVHD on day + 180. Mycophenolate mofetil 15 mg/kg was administered every 12 h from day − 3 to day + 30. MTX 15 mg/m^2^ was administered on day + 1 and reduced to 10 mg/m^2^ on days + 3, + 5, and + 11 [20]. Regarding morphological evaluations, flow cytometry and quantitative measurements of MLL-r by qRT-PCR in bone marrow (BM) samples were performed at scheduled time points to detect minimal residual disease (MRD) (within two weeks before the start of the preparative regimen; 1, 2, 3, 4, 5, 6, 9, and 12 months post-HSCT, then every 6 months thereafter).

### Definitions

Complete remission (CR) was defined as the detection of < 5% bone marrow blasts without any localized leukemic infiltrates. MRD-positive (MRD +) status was defined as CR with the presence of MLL rearrangements detected on qRT-PCR and/or positive expression (≥ 10^–4^ myeloblasts) of MRD detected via flow cytometry. MRD-negative (MRD-) status was defined as CR with negativity for MLL rearrangements by qRT-PCR and negative expression (< 10^–4^ myeloblasts) of MRD detected via flow cytometry. Relapse was defined as the detection of ≥ 5% bone marrow blasts and/or extramedullary leukaemia. Clinical graft-versus-host disease (GVHD) occurring prior to 100 days after HSCT is called acute GVHD (aGVHD), and that occurring after 100 days is called chronic GVHD (cGVHD). The major organs involved in acute GVHD were the skin, gastrointestinal tract, and liver. The diagnosis of GVHD was in accordance with the common international criteria [21, 22].

### Statistical analysis

OS was defined as the time from transplantation to death or last follow-up, and EFS was defined as the time from transplantation to the first event (relapse at any site, death, or secondary malignancy) or last follow-up. Non-relapse mortality (NRM) was defined as death without prior relapse or progression. The cumulative incidence of relapse (CIR) was calculated from the date of CR to the first relapse. Considering the competing risks of death and relapse, competing risk analysis was performed to assess the cumulative incidence. The chi-square test or Fisher’s exact test were used to estimate significant differences between group comparisons. Kaplan–Meier methods and the log-rank test were used to estimate and compare OS and EFS. All significant prognostic factors in a univariate analysis with *P* < 0.1 were included in a multiple Cox regression model. Data analysis was performed using SPSS 22.0 (IBM, Armonk, NY) and R version 3.5.3 (R Foundation for Statistical Computing, Vienna, Austria). The level of statistical significance was set at *P* < 0.05.

## Results

### Patient characteristics

A total of 44 paediatric patients were included in our study between January 2014 and October 2019. Table 1 summarises the patients’ characteristics. The population included 19 (43.1%) female and 25 (56.8%) male patients, with a median age of 9.0 (range, 1.1–17.0) years. The most common FAB classification was M5. The most frequent MLL-r subtype was t(9;11)(p22;q23)/AF9 (22.7%), followed by MLL-partial tandem duplication (PTD) (18.2%), t(10;11)(p13;q23)/AF10 (15.9%), t(11;19)(q23;p13.1)/ELL (11.4%), t(6;11)(q27;q23)/AF6 (9.1%), t(1;11)(q21;q23) /AF1Q (6.8%) and t(11;19)(q23;p13.3)/ENL (4.5%). Twenty-seven (61.4%) patients achieved CR after the first cycle of induction. Twelve (27.3%) patients achieved CR after the second cycle of induction, and 5 (11.4%) patients achieved CR after the third cycle of chemotherapy. All the patients were in CR1 at the time of HSCT. The median interval between diagnosis to allo-HSCT was 4 months (range, 3–7 months). In total, 37 (84.1%) patients underwent haplo-HSCT.

**Table 1:** Main characteristics of the 44 paediatric patients with MLL-r AML undergoing HSCT

Items	No. patients	Percentage
Age(years)		
Median range	9.0(1.1–17.0)	
< 10	24	54.5%
≥ 10	20	45.5%
Sex		
Female	19	43.2%
Male	25	56.8%
FAB subtype		
M2	6	13.6%
M4	7	15.9%
M5	27	61.4%
M6	2	4.5%
M7	2	4.5%
MLL-r subtype		
t(9;11)(p22;q23)/AF9	10	22.7%
MLL-PTD	8	18.2%
t(10;11)(p13;q23)/AF10	7	15.9%
t(11;19)(q23;p13.1)/ELL	5	11.4%
t(6;11)(q27;q23)/AF6	4	9.1%
t(1;11)(q21;q23)/AF1q	3	6.8%
t(11;19)(q23;p13.3)/ENL	2	4.5%
other MLL-r	5	11.4%
Additional gene abnormalities		
EVI1	7	15.6%
FLT3-ITD	8	17.8%
Risk stratification		
High risk	35	79.5%
Intermediate risk	9	20.5%
Cycles of induction to achieve CR		
1	27	61.4%
2	12	27.3%
3	5	11.4%
MRD- after 2 cycles of induction		
Yes	23	52.3%
No	21	47.7%
CNS infiltration		
Yes	7	15.9%
No	37	84.1%
Donor source		
HID	37	84.1%
Non-HID	7	15.9%
MUD	1	
MSD	6	
Donor gender		
Male	36	81.8%
Female	8	18.2%
Donor age(Median range)	33.0(6.0–45.0)	
Relationship between donor and recipient		
Father	31	70.5%
Mother	5	11.4%
Brother	4	9.1%
Sister	3	6.8%
Unrelated	1	2.3%
Source of stem-cells		
BM and Pb	43	97.7%
Pb	1	2.3%
Median mononuclear cells × 10^8^/kg(range)	9.42(3.1–16.1)	
Median CD34 + counts × 10^6^/kg(range)	2.61(0.76–10.7)	

*MLL-r* MLL rearrangement, *CNS* central nervous system, *HID* haploidentical donors, *MUD* matched unrelated donor, *MSD* matched sibling donor, *BM* bone marrow, *Pb* peripheral blood, *PTD* partial tandem duplication, *EVI1* ecotropic viral integration site 1, *FLT3-ITD* fms-like tyrosine kinase 3 internal tandem duplication

### Outcomes

#### Engraftment and GVHD

One patient died on the eighth day after transplantation due to intracranial infection without neutrophil and platelet engraftment. Another patient died on the thirty-ninth day after transplantation due to severe GVHD without platelet engraftment. Thus, 43 patients achieved neutrophil engraftment with a median time to engraftment of 12 (range, 8–23) days, and 42 patients achieved platelet engraftment with a median time to engraftment of 16 (range, 8–85) days. For all the 44 patients, the 100-day cumulative incidence of grade II–IV aGVHD was 27.3% ± 6.7% (95% confidence interval [CI]: 15.1%–41.0%), and that of grade III–IV aGVHD was 15.9% ± 5.5% (95% CI: 6.9%–28.2%). As of the last follow-up, 42 patients survived for more than 100 days after HSCT. The overall 3-year cumulative incidence of cGVHD was 40.8% ± 8.2% (95% CI: 24.5%–56.5%), and that of extensive cGVHD was 16.7% ± 6.9% (95% CI: 5.8%–32.3%). Meanwhile, for the 37 patients who received haplo-HSCT, the 100-day cumulative incidence of grade II–IV aGVHD and grade III–IV aGVHD was 32.4% ± 7.7% (95% CI: 18.1%–47.7%) and 18.9% ± 6.4% (95% CI: 8.2%–33.0%), respectively. The 3-year cumulative incidence of cGVHD and extensive cGVHD was 45.2% ± 8.8% (95% CI: 27.3%–61.6%) and 18.7% ± 7.6% (95% CI: 6.5%–35.7%). In contrast, for the remaining 7 patients who received HLA-matched transplants, only one patient developed grade I aGVHD.

#### Relapse and NRM

The median follow-up period was 27.0 (range, 0.3–82) months. Up to the last follow-up date (December 21, 2020), 11 patients experienced relapse at a median of 11 (range, 3–33) months after HSCT. Nine patients had only a haematologic relapse, while two patients had an extramedullary relapse, followed by a haematologic relapse. Of them, six received chemotherapy plus donor lymphocyte infusion (DLI), two received DLI only, one received chemotherapy plus secondary transplantation, one received chemotherapy only, and one abandoned further therapy. During the follow-up period, 12 patients died. The causes of death included relapse (*n* = 8) and NRM (*n* = 4). Of the 4 patients who died of NRM: two died of infection, one of aGVHD, and one due to suicide.

#### OS, EFS, and CIR

**Fig. 1:**
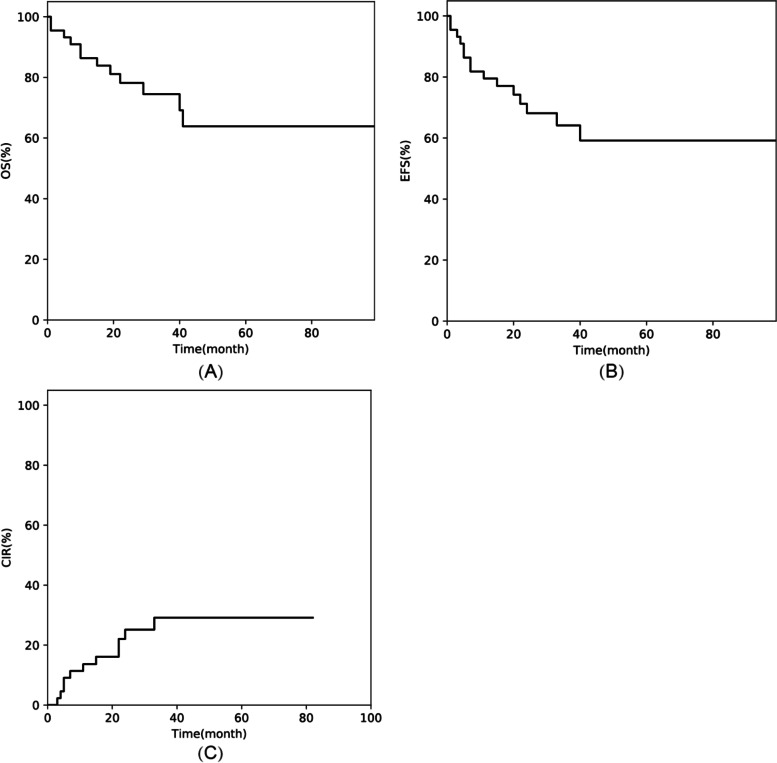
Outcomes of 44 paediatric patients with MLL-r AML undergoing HSCT: (**A**) overall survival (OS), (**B**) event-free survival (EFS), and (**C**) cumulative incidence of relapse (CIR)

**Fig. 2:**
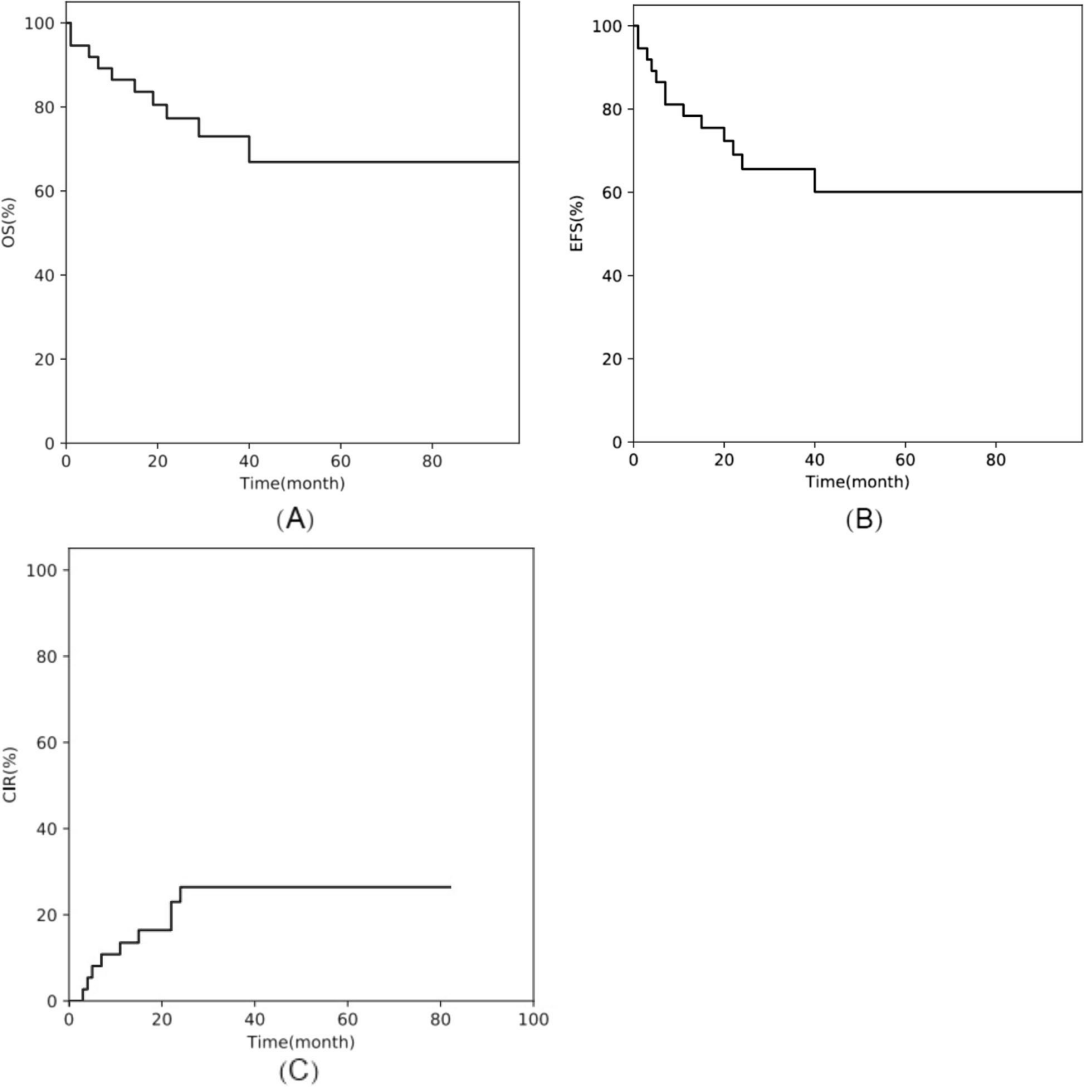
Outcomes of 37 paediatric patients with MLL-r AML undergoing haplo-HSCT: (**A**) overall survival (OS), (**B**) event-free survival (EFS), and (**C**) cumulative incidence of relapse (CIR)

For all the 44 patients, the 3-year OS, EFS, and CIR were 74.5% ± 7.2% (95% CI, 57.2%–85.6%), 64.1% ± 8.0% (95% CI: 46.3%–77.4%), and 29.1% ± 7.7% (95% CI: 15.1–44.8%), respectively (Fig. 1). Meanwhile, for the 37 patients who received haplo-HSCT, the 3-year OS, EFS, and CIR were 73.0% ± 7.9% (95% CI: 53.9%–85.2%), 65.6% ± 8.2% (95% CI: 47.1%–78.9%), and 26.4% ± 7.7% (95% CI: 19.8–42.2%), respectively (Fig. 2).

#### Impact of MRD status at pre-HSCT

In total, 29 patients were MRD- (MRD- pre-HSCT cohort), and 15 patients were MRD + (MRD + pre-HSCT cohort) pre-HSCT. The two cohorts had similar baseline characteristics (Table 2).

**Table 2:** Patient characteristics for MRD + cohort and MRD- cohort

Items	MRD + pre-HSCT	MRD- pre-HSCT	P
	(*n* = 15)	(*n* = 29)	
Age(years)			
Median range	11.0(1.1—17.0)	7.0(1.2—17.0)	0.21
≥ 10	9	11	
< 10	6	18	
Sex			0.759
Female	7	12	
Male	8	17	
AF9			1
No	12	22	
Yes	3	7	
WBC(× 10^9^/L)			0.333
≦50	9	14	
> 50	4	13	
NA	2	2	
CNS infiltration			0.675
No	12	25	
Yes	3	4	
1 cycle to CR			0.198
No	8	9	
Yes	7	20	
Haplo-HSCT			0.393
No	1	6	
Yes	14	23	

**Fig. 3:**
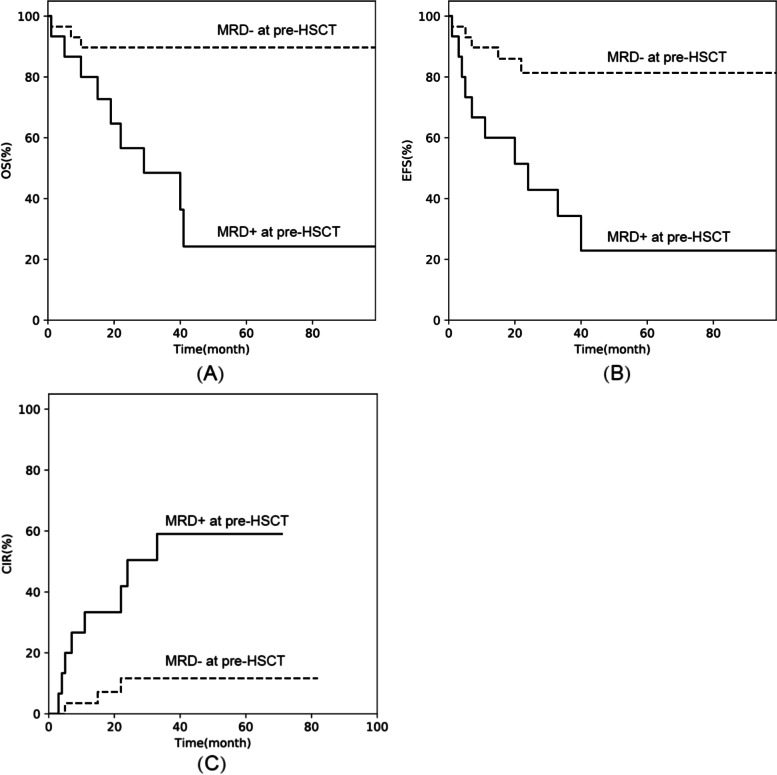
Outcomes of the MRD- and MRD + at pre-HSCT cohorts: (**A**) overall survival (OS), (**B**) event-free survival (EFS), and (**C**) cumulative incidence of relapse (CIR)

The survival difference between the two cohorts was significant. The 3-year OS was 89.7% ± 5.7% (95% CI: 71.3%–96.5%) in the MRD- pre-HSCT cohort and 48.5% ± 13.8% (95% CI: 20.9%–71.6%) in the MRD + pre-HSCT cohort (*p* = 0.001). Meanwhile, the 3-year EFS was 81.4% ± 7.6% (95% CI: 60.9%–91.9%) in the MRD- pre-HSCT cohort and 34.3% ± 13.3% (CI: 11.3%–59.2%) in the MRD + pre-HSCT cohort (*p* = 0.001). The estimated 3-year CIR rate was significantly lower (*p* = 0.001) in the MRD- pre-HSCT cohort (11.7% ± 6.4%, 95% CI: 2.8%–27.7%) than in the MRD + pre-HSCT cohort (59.0% ± 14.9%, 95% CI: 26.0%–81.4%) (Fig. 3).

#### Risk factors for relapse, OS and EFS

The univariate analyses revealed that MRD + at pre-HSCT, II–IV aGVHD, and one cycle to CR were significant factors affecting OS. MRD + at pre-HSCT, II–IV aGVHD, one cycle to CR, and central nervous system (CNS) infiltration were also significant factors that influenced EFS and CIR (Table 3).

**Table 3:** Univariate analysis of factors associated with long-term outcomes (*n* = 44)

Items	N	3-year-OS(%)	P	3-year-EFS(%)	P	3-year-CIR	P
Sex			0.954		0.643		0.643
Female	19	77.2% ± 10.2%		66.2% ± 11.4%		23.9% ± 10.6%	
Male	25	72.3% ± 10.0%		63.0% ± 10.9%		33.0% ± 10.7%	
WBC(× 10^9^/L)			0.865		0.512		0.527
< 50	23	82.6% ± 7.9%		70.7% ± 10.4%		25.0% ± 9.9%	
≥ 50	17	66.8% ± 15.0%		57.0% ± 13.9%		31.2% ± 13.2%	
Haplo-HSCT			0.87		0.881		0.881
No	7	85.7% ± 13.2%		42.9% ± 31.0%		57.1% ± 31.0%	
Yes	37	73.0% ± 7.9%		65.6% ± 8.2%		26.4% ± 7.7%	
Age(years)			0.131		0.52		0.532
< 10	24	87.3% ± 6.9%		64.5% ± 11.9%		31.4% ± 11.6%	
≥ 10	20	60.0% ± 12.0%		61.4% ± 11.8%		28.6% ± 11.1%	
MRD + at pre-HSCT			0.001		0.001		0.001
No	29	89.7% ± 5.7%		81.4% ± 7.6%		11.7% ± 6.4%	
Yes	15	48.5% ± 13.8%		34.3% ± 13.3%		59.0% ± 13.8%	
CNS infiltration			0.175		0.087		0.087
No	37	78.3% ± 7.5%		68.1% ± 8.7%		23.9% ± 8.2%	
Yes	7	57.1% ± 18.7%		42.9% ± 18.7%		57.1% ± 18.7%	
AF9			0.312		0.234		0.233
No	34	78.5% ± 8.0%		68.7% ± 9.1%		25.6% ± 8.7%	
Yes	10	60.0% ± 15.5%		50.0% ± 15.8%		40.0% ± 15.5%	
EVI1 Expression			0.612		0.969		0.949
No	36	71.4% ± 8.4%		63.9% ± 8.5%		27.8% ± 8.0%	
Yes	8	87.5% ± 11.7%		70.0% ± 18.2%		30.0% ± 18.2%	
FLT3-ITD mutations			0.737		0.392		0.39
No	37	75.9% ± 7.6%		65.9% ± 8.7%		28.8% ± 8.4%	
Yes	7	68.6% ± 18.6%		57.1% ± 18.7%		28.6% ± 17.1%	
1 cycle to CR			0.100		0.004		0.003
No	17	64.2% ± 11.8%		37.3% ± 12.5%		50.9% ± 12.9%	
Yes	27	80.7% ± 90.0%		82.6% ± 8.1%		13.7% ± 7.5%	
MRD- after 2 cycles			0.305		0.199		0.205
No	21	70.2% ± 10.2%		54.9% ± 11.3%		40.4% ± 11.2%	
Yes	23	82.6% ± 7.9%		77.4% ± 8.9%		13.9% ± 7.5%	
II-IV aGVHD			0.076		0.012		0.011
No	32	83.2% ± 6.9%		74.7% ± 8.6%		22.3% ± 8.3%	
Yes	12	43.8% ± 20.2%		37.5% ± 15.3%		45.8% ± 15.7%	
cGVHD			0.187		0.442		0.428
No	28	69.5% ± 9.2%		61.0% ± 10.0%		28.3% ± 9.3%	
Yes	16	83.3% ± 10.8%		70.0% ± 12.9%		30.0% ± 12.9%	

*OS* overall survival, *EFS* event-free survival, *CIR* cumulative incidence of relapse, *WBC* white blood cell, *Haplo-HSCT* haploidentical haematopoietic stem cell transplantation, *MRD* + Minimal Residual Disease-positive, *CNS* central nervous system, *aGVHD* Acute graft versus host disease, *cGVHD* Chronic graft versus host disease, *NA* not available, *EVI1* ecotropic viral integration site 1, *FLT3-ITD* fms-like tyrosine kinase 3 internal tandem duplication

In the multivariate analysis, MRD + at pre-HSCT was an independent risk factor for OS, EFS, and CIR. Meanwhile, III–IV aGVHD and one cycle to CR were independent risk factors for EFS (Table 4).

**Table 4:** Multivariate analysis of factors associated with long-term outcomes (*n* = 44)

Items	OS		EFS		CIR	
	HR(95%CI)	P	HR(95%CI)	P	HR(95%CI)	P
MRD + at pre-HSCT	5.83(1.52–22.29)	0.01	4.47(1.48–13.48)	0.01	6.44(1.61–25.74)	0.01
II-IV aGVHD	2.39(0.69–8.25)	0.17	3.57(1.08–11.8)	0.04	3.13(0.70–13.95)	0.13
1 cycle to CR	0.61(0.18–2.02)	0.42	0.29(0.09–0.97)	0.04	0.24(0.05–1.05)	0.06
CNS infiltration			1.32(0.33–5.31)	0.69	2.51(0.50–12.62)	0.26

*OS* overall survival; EFS: event-free survival, *CIR* cumulative incidence of relapse, *MRD* + Minimal Residual Disease-positive, *aGVHD* Acute graft versus host disease, *CNS* central nervous system

## Discussion

According to previous studies, the presence of MLL-r in AML is a poor prognostic predictor. Therefore, it is essential to evaluate the efficacy of allo-HSCT in such cases. According to the age distribution of MLL-r, most of the patients are infants (primarily acute lymphoblastic leukaemia) and young-to-middle-aged adults (primary AML) [23]. Thus, most studies have focused on infants and adults. To the best of our knowledge, this study is one of the largest series of non-infant children with MLL-r AML treated with allo-HSCT. Our results demonstrate that allo-HSCT (especially haplo-HSCT) in CR1 may improve the prognosis of non-infant children with MLL-r AML, and pre-HSCT MRD status affected the outcomes significantly.

In adult patients with MLL-r AML, several studies have confirmed that allo-HSCT is more beneficial than chemotherapy alone [8, 9, 24]. In contrast, the effectiveness of HSCT remains controversial for paediatric patients with MLL-r AML. Balgobind et al. [6] have conducted the largest such study, which involved 756 paediatric patients. They reported that HSCT did not improve the prognosis. Miyamura et al. [11] reported that the 3-year OS and EFS of 90 paediatric patients with MLL-r AML after allo-HSCT were 52.1% and 46.7%, respectively. These values were significantly lower than the ~ 70% overall long-term survival in paediatric AML. However, Wang-Yu et al. [10] reported that 56 patients with MLL-r AML (10 children and 46 adults) who received allo-HSCT had a 3-year OS of 61.8%; the adults and children had comparable OSs and leukaemia-free survivals. In contrast, all the 29 patients who did not receive allo-HSCT relapsed. Therefore, these study findings imply that allo-HCT should be recommended for all patients with MLL-r AML in CR1. In our study, the 3-year OS of 74.5% and EFS of 64.1% for the 44 paediatric patients were higher than previously reported rates. Several factors may have contributed to these favourable outcomes. With the continuous advances in allo-HSCT, the long-term survival of paediatric patients with MLL-r after allo-HSCT is improving. In our study, most of the patients were treated in the last five years, which is different from prior studies that included patients from 15–20 years ago. Furthermore, recent studies have demonstrated that patients receiving HSCT in CR1 have a better prognosis than those beyond CR1 [9–12]. All the patients enrolled into our cohort had achieved CR1 at pre-HSCT, further increasing the long-term survival rates.

Numerous studies have shown that the presence of MRD at pre-HSCT has a very strong impact on outcomes [25–27]. A recent meta-analysis showed that being MRD + at pre-transplant was associated with worse LFS, OS, and CIR [28]. Konuma et al. [29] assessed the MRD status of 109 patients with MLL-r AML at pre-HSCT, with a probability of a 3-year OS of 39% in the MRD + cohort and 64% in the MRD- cohort (*P* = 0.02). In fact, our research showed that MRD + patients at pre-HSCT had a worse survival and higher risk of relapse than the MRD- patients, and confirmed that pre-HSCT MRD + was an independent risk factor for OS, EFS, and CIR. Therefore, strategies to achieve an MRD-negative status at pre-HSCT are very necessary. MRD + patients at pre-HSCT tend to be relatively insensitive to chemotherapy. Extra consolidation therapy may not reverse an MRD-positive state into an MRD-negative state; however, it is associated with long-term adverse effects and a high rate of treatment-related mortality. In this era of targeted therapies, we expected to identify therapeutic agents that specifically target MLL fusion proteins and/or the downstream pathways these fusions dysregulate. Currently, inhibitors against the kinase P-TEFb (CDK9/CyclinT1) [30], histone methyltransferases DOT1L [31], and bromodomain and extra-terminal domain family of proteins [32] are undergoing clinical testing for AML, and inhibitors of the MENIN-MLL interaction have been described and are undergoing pre-clinical evaluation [33, 34]. Presently, whether MRD + patients at pre-HSCT should receive maintenance therapy after HSCT remains controversial. Given the high risk of disease recurrence, Yi Zhou et al. [26] suggested that MRD + patients at pre-HSCT should receive pre-emptive therapeutic strategies after HSCT. Accumulating evidence has confirmed that high-risk AML patients might benefit from maintenance therapy post-transplantation. The initiation of directed agents in patients with identified mutations (e.g., FLT3 and Philadelphia chromosome) prior to allo-HCT, have generally better post-HCT outcomes [35, 36]. Lei Gao et al. [37] reported that minimal-dose decitabine maintenance combined with recombinant human granulocyte colony stimulating factor after allo-HSCT can reduce the relapse rate in high-risk AML patients undergoing allo-HSCT, with a 2-year relapse rate of 15.0% and 38.3% in the intervention and non-intervention groups, respectively. Chapuis et al. [38] reported that 12 high-risk patients (MRD + pre-HSCT or high-risk cytogenetic/molecular features) received T cell receptor gene therapy targeting WT1 as post-transplant maintenance had a 100% relapse-free survival, while the control group of 88 patients had a 54% relapse-free survival (*P* = 0.002). However, the maintenance strategies may potentially increase the NRM risk because of residual toxicity from HCT, the fragility of the newly transplanted marrow and the existence of other transplant-related complications. Therefore, there is an urgent need to identify an effective and safe approach.

Notably, 37 patients (84.1%) in our cohort received haplo-HSCT. Previous studies showed that haplo-HSCT had a higher incidence of GVHD than HLA-matched transplants [39]; our results were in line with previous reports. However, haplo-HSCT also had certain strengths. Our previous study showed that the outcomes of haplo-HSCT are equivalent to those of MSDT and MUDT [40], and haplo-HSCT may even be superior in eradicating pre-transplantation MRD [41]. Yu et al. [42] assessed 189 patients with high-risk AML who were divided into an HID cohort (n = 83) and MSD cohort (n = 106). The cumulative incidence of post-MRD + was significantly lower in the HID groups than in the MSD groups (18% vs. 42%, p < 0.001). It was considered that HID might have a stronger graft-versus-leukaemia effect than MSD. In our study, III-IV aGVHD was a poor prognostic factor for EFS, but with no observed significant difference in OS in the multivariate analysis. We speculated that the adverse effect of high GVHD may have been offset by the benefits of a stronger graft-versus-leukaemia effect of HID.

The major limitations of this study were its retrospective design, single-centre design, and limited sample size. Previous reports have indicated that AML with t(9,11) is associated with a better prognosis than other 11q23 abnormalities [6], and the expression of ecotropic viral integration site 1(EVI1) or fms-like tyrosine kinase 3-internal tandem duplication (FLT3-ITD) mutation was a poor prognostic factor [23, 43]. The factors in this study were not significant. This may be related to the small number of cases. Thus, our results need to be further verified in multi-centre and large-scale studies.

## Conclusions

In conclusion, our study demonstrates the superior outcomes of HSCT for non-infant children with MLL-r AML and suggests that allo-HSCT (especially haplo-HSCT) in CR1 may improve the prognosis of these patients. Pre-HSCT MRD status was the most significant prognostic factor for relapse and survival. Therefore, allo-HSCT can be a viable strategy in these patients, and close monitoring of MRD at pre-HSCT is crucial.

## Data Availability

The datasets used and analysed during the current study are available from the corresponding authors on reasonable request.

## Abbreviations

aGVHD: Acute graft-versus-host disease
allo-HSCT: Allogeneic haematopoietic stem cell transplantation
AML: Myeloid leukaemia
ATG: Anti-thymocyte globulin
BM: Bone marrow
CIR: Cumulative incidence of relapse
CIR: Cumulative incidence of relapse
CNS: Central nervous system
CR: Complete remission
CR1: Complete remission
CSA: Cyclosporine A
EFS: Event-free survival
EVI1: Ecotropic viral integration site 1
FISH: Fluorescence in situ hybridisation
FLT3-ITD: Fms-like tyrosine kinase 3-internal tandem duplication
GVHD: Graft-versus-host disease
HIDs: Haploidentical donors
HLA: Human leukocyte antigen
MRD: Minimal residual disease
MLL-r: Mixed-lineage leukaemia rearrangement
MMF: Mycophenolate mofetil
MSDT: Matched sibling donor transplant
MTX: Methotrexate
MUDT: Matched unrelated donor transplant
NRM: Non-relapse mortality
OS: Overall survival
qRT-PCR: Quantitative real-time polymerase chain reaction
